# Supplementary material for: Application of flexible parametric cure model in determination of effective factors on the technique of survival peritoneal dialysis patients in adults of Iran
Source: PLoS One. 2025 Jul 22;20(7):e0326748. doi: 10.1371/journal.pone.0326748 (PMC12282859; doi:10.1371/journal.pone.0326748)
Supplement: Attachment — Submitted filename: Review Comments to the Author_final.docx [file pone.0326748.s001.docx]

**Reviewer 1:**

**Minor comments on the abstract:**

We sincerely thank the reviewer for their insightful comments regarding the definition of the event of interest and its implications for the flexible parametric cure model.

1. The term odds ratios (OR) needs to be changed to hazard ratios (HR) in the result section since a Cox-Proportional Hazards model was utilized. Edited
2. ESR typically stands for Erythrocyte Sedimentation Rate. You have used the short form for Inflammatory Index Rate. Please clarify. Edited

**Methods/Results:**
1. What was the pharmacoepidemiologic study design employed for this study? (Cohort, Case-control, nested case-control etc.). retrospective (historical) cohort

2. How did you define the baseline (pre-index) and follow-up (post-index) periods? What was the index event? Was it receipt of first PD in the registry?

In our study, the index event was defined as the initiation of peritoneal dialysis (PD), which corresponds to the first recorded PD treatment in the Iranian PD registry. This is consistent with standard practices in survival analysis of dialysis patients, where the start of dialysis is typically used as the index event.

Baseline (pre-index) period: The baseline period was defined as the time prior to the initiation of PD. During this period, demographic, clinical, and laboratory data were collected from the registry. These data were used to characterize the patients at the start of PD and to assess potential predictors of survival. For example, laboratory values such as hemoglobin, creatinine, and albumin were recorded as the most recent measurements prior to PD initiation.

Follow-up (post-index) period: The follow-up period began at the initiation of PD (index event) and continued until the occurrence of the event of interest (discontinuation of PD due to technique failure or death) or censoring (e.g., switching to hemodialysis or being placed on the kidney transplant emergency list). Patients were followed until the end of the study period (February 2018) or until they were lost to follow-up.

3. The event of interest, discontinuation of PD has two types of patients; (1) patients who died and (2) patients who discontinued PD. Are both these events considered “uncured” patients in your flexible parametric model? If yes, it would impact your “uncured or susceptible” estimates since patients who discontinued PD may not necessarily die and could very well shift to hemodialysis, transplant or other options. In this case, they would get censored which is not appropriate to your grouping of interest since you want them in the “uncured or susceptible” group. As a result, a disproportionately higher number of patients would get censored as seen in the results section. Is there a better way to define the outcome? How did you define discontinuation of PD as an outcome in the database?

We acknowledge the reviewer's concern regarding the potential impact of discontinuation of peritoneal dialysis (PD) for reasons other than death on the estimation of the "uncured" or "susceptible" population in the flexible parametric cure model.

**To address this concern, we would like to clarify the following:**

In our study, we defined the event of interest as the discontinuation of PD, which encompasses two primary scenarios:

Death: Patients who died while on PD were classified as having experienced the event of interest. This definition aligns with the clinical objective of assessing long-term survival in PD patients, as death serves as the most definitive endpoint.

Transition to Other Therapies: Patients who transitioned to hemodialysis or were placed on the kidney transplant emergency list were considered censored. This decision was made to ensure that patients who were still receiving care but had changed modalities were not mistakenly classified as having failed PD.

We recognize that censoring patients who transitioned to hemodialysis or were placed on the transplant list could impact our "uncured" or "susceptible" estimates. However, our rationale for this classification is based on the following:

Clinical Relevance: Transitioning to hemodialysis or being listed for a transplant indicates ongoing medical management and does not necessarily reflect a failure of PD. These patients may still be at risk of adverse outcomes, but they are not classified as having "failed" PD in the traditional sense.

Data Integrity: By censoring these patients, we aimed to maintain the integrity of our survival analysis, focusing on those who experienced the event of interest (death) while on PD.

However, the use of a flexible parametric survival model allowed us to estimate survival rates while accounting for the complexities of our dataset.

4. Please rearrange the Maller and Zhou test explanation from the results to the methods section to test the following assumptions: (1) presence of patients with long-term survival; (2) sufficient duration of the study period.

**I will place the content in the methods section and, based on your comments, I made the necessary edits, which are as follows:**

Before using the cure model, the assumptions of the model were carefully examined. These assumptions include the presence of patients with long-term survival and sufficient duration of the study period, which were tested using statistical tests.

At first, the presence of immune individuals was first visually assessed using Kaplan-Meier estimates. In datasets with immune individuals, censored observations are typically observed at longer time points on the right side of the Kaplan-Meier survival curve. A flattening of the curve at a value greater than zero suggests the presence of both susceptible and immune individuals.

Statistical tests were used then to examine the proportion of individuals whose censoring time exceeds the largest event occurrence time. The question arises as to whether a significant percentage of individuals have long-term survival or not. In some cases, clinical experiences and biological evidence can demonstrate that a proportion of cured individuals exists. Otherwise, using statistical tests can be helpful. This hypothesis can be written as $H_{0}:p=1$, meaning that under the assumption $H_{0}$, all individuals are at risk of the event occurring, and there is no proportion of cured individuals. In the proposed method by Maller and Zhou, a test based on the i.i.d censoring model with the assumption of exponential and uniform censoring distribution is presented. Critical values for accepting or rejecting the existence of a proportion of cured individuals have been compiled in a table. This table is based on the censoring model and the uniform and exponential censoring distribution. To use this table, the estimated number of susceptible individuals is first calculated from the sample, and based on that, the appropriate censoring distribution is computed. Then, the critical value is extracted from the table, considering the sample size at a specified significance level, and based on that, the hypothesis of the existence of patients with long-term survival is accepted or rejected (1).

After obtaining sufficient evidence regarding the existence of patients with long-term survival, the second point is to ensure that the significance of the proportion of cured individuals and its difference from zero is not due to the short follow-up duration. For this purpose, Maller and Zhou have developed tables under the conditions of the previous test (i.i.d censoring times and uniform or exponential censoring distribution) through simulation. The following steps are carried out:

1- Calculate the last observed failure time $t_{(n)}^{*}$

2- Calculate the last observed censoring time $t_{n}$

3- Compute $\delta_{n}=t_{n}-t_{(n)}^{*}$

4- Count the number of failures in the interval $N_{n}-(2t_{n}^{*}-t_{n}, t_{(n)}^{*}]$

5- Calculate $q_{n}=\frac{N_{n}}{n}$

The obtained value of $q_{n}$ is compared with the critical value related to the sample size of the study and B (U[0,B]) or $\mu$ (Exp($\mu$)) from the simulation tables of Maller and Zhou (2).

### ****1. Testing for the Presence of Immune or Cured Individuals in Censored Survival Data****

Maller, R. A., & Zhou, S. (1995). Testing for the presence of immune or cured individuals in censored survival data. Biometrics, 51(1), 280–284.

<https://www.jstor.org/stable/2533330>.

### ****2. Testing for Sufficient Follow-Up****

1- Maller, R. A., & Zhou, S. (1992). Testing for sufficient follow-up and outliers in survival data. Journal of the American Statistical Association, 87(420), 1499-1506.

<https://doi.org/10.1080/01621459.1992.10476282>

5. Is a backward variable selection model the most appropriate approach for identifying baseline risk factors? A backward selection model may tend to eliminate potential risk factors that are clinically meaningful but that aren’t statistically significant in your case at p<0.2. In such cases it would be better to rely on expert opinion. Furthermore, some of the clinically meaningful variables that get excluded as a result of a lack of statistical significance may have a potential interaction with other variables. Did the study account for any covariate interactions?

Below, I will address your concerns and clarify our approach.

We acknowledge that a backward variable selection model can potentially exclude clinically meaningful variables that are not statistically significant at the p<0.2 level. In our study, we aimed to balance statistical rigor with clinical relevance. While we primarily relied on statistical criteria for variable selection, we also consulted with clinical experts to ensure that important risk factors were not overlooked.

To further validate our approach, we conducted a sensitivity analysis using forward selection and full model approaches (including all candidate variables regardless of statistical significance). The results of these analyses were consistent with our primary findings, suggesting that the backward selection process did not introduce significant bias.

We appreciate your comments and would like to address the points raised regarding the handling of interaction effects and the use of splining in our model.

The flexible cure model is already a complex statistical framework designed to account for the presence of long-term survivors, often referred to as immune individuals. This model is particularly useful in survival analysis, where it aims to distinguish between those who will experience the event of interest (e.g., death, disease recurrence) and those who will not, thereby modeling the survival function more accurately. One of the key features of this model is its utilization of spline-based approaches, which enhance its ability to capture intricate patterns in the data. Splining allows for modeling relationships in a piecewise manner, effectively capturing changes in the relationship between variables at different levels. This can be thought of as examining the effect layer by layer, where each segment of the spline can represent different behaviors or trends in the data. For instance, when analyzing the impact of time (year) on survival outcomes, splines can adapt to shifts in trends that may occur at various points in time. By using splines, we can model non-linear relationships between a predictor (like year) and the outcome, which can vary depending on another variable (like age). This capability is crucial in many real-world scenarios where the relationship between variables is not strictly linear. While splines do not explicitly model interactions in the same way as traditional interaction terms, they can still account for varying effects of one variable (e.g., year) across levels of another variable (e.g., age). This means that the effect of year on the outcome can change depending on age, similar to how an interaction term would function and splines can capture this nuanced relationship without the need for complex interaction terms.

Additionally, this approach provides significant flexibility in capturing complex relationships within the data. By allowing for different slopes and intercepts at various levels of the predictors, splines can uncover hidden patterns that might be overlooked with simpler modeling techniques.

6. Please specify additional details regarding what type of a parametric cure model was utilized for this study? Were restricted cubic splines used? Did the model account for excess hazard in addition to expected hazard to calculate the overall hazard function? In the flexible parametric cure model, did the authors model log hazard or log cumulative hazard?

1- Traditional parametric survival models, such as the exponential, Weibull, and Gompertz models, impose strong assumptions about the shape of the baseline hazard function. For example:

- The **exponential model** assumes a constant hazard over time.
- The **Weibull model** assumes a monotonically increasing or decreasing hazard.
- The **Gompertz model** assumes an exponential increase or decrease in the hazard.

These assumptions are often too restrictive for real-world data, where the hazard function may have a more complex shape. To address this limitation, we employed a **flexible parametric survival model** based on the approach proposed by Royston and Parmar (reference 46). The flexible parametric cure model is particularly well-suited for survival data with a plateau in the survival curve, as it allows for the estimation of both the proportion of cured individuals and the survival function for uncured individuals.

2. Use of Restricted Cubic Splines

Yes, restricted cubic splines were used to model the baseline hazard function. Restricted cubic splines are piecewise polynomial functions of degree 3 that are constrained to be linear beyond the boundary knots. This constraint ensures that the splines are smooth and continuous while reducing the influence of extreme values in the tails of the distribution. The use of restricted cubic splines provides several advantages:

Flexibility: They can capture complex, nonlinear shapes in the hazard function without imposing restrictive parametric assumptions.

Smoothness: They ensure that the hazard function is smooth and continuous, which is important for interpretability.

Stability: The linear constraint beyond the boundary knots reduces the risk of overfitting and improves the stability of the model.

3- **Flexible parametric survival model:**

The flexible parametric survival model relative survival is fit on log cumulative-excess-hazard- scale by using restricted cubic splines to estimate the baseline cumulative excess hazard. By integrating $h\left( t \right)=h^{*}\left( t \right)+\lambda(t)$, we get $H\left( t \right)=H^{*}\left( t \right)+\Lambda(t)$ where $H\left( t \right)$ is overall cumulative hazard, $H^{*}\left( t \right)$ is the expected cumulative hazard and $\Lambda(t)$ is the cumulative excess hazard. We model the cumulative excess hazard on the log scale by using restricted cubic splines:

$$ln\{\Lambda\left( t \right)\}=ln\{-\ln R(t)\}=\gamma_{00}+\gamma_{01}v_{1}\left( x \right)+\gamma_{02}v_{2}\left( x \right)+\ldots+\gamma_{0K-1}v_{k-1}\left( x \right)$$

where x=ln(t), K is the number of knots, $v_{1}\left( x \right)=x$ , and for j = 2, …, K-1, the *j*th basis function is defined as

$$v_{j}\left( x \right)=(x-k_{j})_{+}^{3}-\lambda_{j}(x-k_{min})_{+}^{3}-(1-\lambda_{j})(x-k_{max})_{+}^{3}$$

Where $u_{+}=u$ if $u>0$ and $u_{+}=0$ if $u\leq0$ , $k_{min}$ is the position of the first knot, $k_{max}$ the position of the last knot, and $\lambda_{j}=\frac{k_{max}-k_{j}}{k_{max}-k_{min}}$

Introducing covariate z:

$$ln\{\Lambda\left( t \right)\}=ln\{-\ln R(t)\}=\gamma_{00}+\gamma_{01}v_{1}\left( x \right)+\gamma_{02}v_{2}\left( x \right)+\ldots+\gamma_{0K-1}v_{k-1}\left( x \right)+\beta^{T}z$$

**Flexible parametric cure models:**

When cure is reached, the excess-hazard rate is 0, and the cumulative excess hazard will be constant after this time. By forcing the log cumulative excess hazard in the flexible parametric survival model not only to be linear but also to have zero slope after the last knot, we can estimate the cure proportion. This is achieved by calculating the spline variable backward (that is, treating the knots in reverse order) and then by restricting the parameter for the linear spline variable to 0 ($\gamma_{01}=0$). The spline basis functions, $v_{j}\left( x \right)$ , are then defined as

$$R(t)=exp(-\exp\left( \gamma_{00}+\gamma_{02}v_{2}\left( x \right)+\ldots+\gamma_{0K-1}v_{k-1}\left( x \right) \right))$$

Which can be written as

$$R(t)=\pi^{exp(\gamma_{02}v_{2}\left( x \right)+\ldots+\gamma_{0K-1}v_{k-1}\left( x \right))}$$

Where $\pi=\exp\left( -exp(\gamma_{00} \right))$.

All spline variables take the value 0 from the point of the last knot, the constant parameter $\gamma_{00}$, is the log cumulative excess hazard at the beyond the last knot for the reference group and therefore be used to predict cure. It is usually preferred to orthogonalize the spline variables, which results in them not being 0 from the point the last knot. Because of this, cure cannot be predicted by a direct transformation of the constant parameters. Therefore, they have been chosen to center the orthogonalized spline variables around the value they at the last knot, which enables direct predictors of cure from the constant parameters. The survival of the uncured can be predicted in the flexible parametric cure model in the same way as the survival of the uncured is predicted in the non-mixture cure model.

4- The flexible parametric cure model in our study was based on the log cumulative excess hazard scale. By modeling the log cumulative hazard, the approach provides greater flexibility in capturing complex hazard shapes over time compared to models based on the log hazard scale.

7. Results lines 157 – 174 need to also be provided in the form of equations. You can include this as supplementary material. The paragraph would be easier for the reader to follow with a set of guiding equations.

Full explanations are provided in Question 4, and all necessary corrections have been made in the manuscript.

8. Should the estimate on line 168 be 0.940 instead of 0.490? Since 0.490 is obtained previously from 1-KM estimator whereas 0.940 has been obtained from exponential distribution. Please verify.

The value of 0.490 has been calculated based on the data. The values of 0.940 and 0.960 are the critical values for the exponential distribution and the uniform distribution, respectively, obtained from the Maller and Zhao table for a sample size of 1000. For larger sample sizes, these critical values approach one, with the value of 0.490 being compared to these values.

9. Figure 2 indicates that the flexible parametric model curve is almost similar to the standard Kaplan-Meier curve but contrastingly different than the other two mixture models. How is using a flexible cure parametric model better than the standard semiparametric Kaplan-Meier model for your study?

Using a flexible parametric model offers several advantages over the standard semiparametric KM model in our study:

1- The flexible parametric model accommodates more complex shapes in the survival function, allowing for varying hazard rates over time. This flexibility can capture underlying patterns in the data that the KM model may miss.

2- Unlike the standard KM model, which primarily focuses on the estimation of survival functions without adjusting for covariates, flexible parametric models can incorporate covariates effectively. This enables a more nuanced understanding of how different factors influence survival.

3- The flexible parametric approach can provide better estimates of survival probabilities at different time points, especially in cases where the data is sparse or where the survival times are heavily censored.

4- Flexible parametric models can extrapolate beyond the observed data, which is particularly useful for predicting long-term survival outcomes. In contrast, the KM curve, being non-parametric, does not allow for extrapolation beyond the last observed event time.

5- Flexible parametric models allow for direct modeling of the hazard function, which can provide insights into the underlying risk patterns over time. The KM model, on the other hand, focuses on the survival function and does not directly model the hazard.

6- The flexible parametric model's similarity to the KM curve, as opposed to the mixture models, suggests that it is a more appropriate choice for our data. Mixture models assume a specific structure (e.g., a mixture of subpopulations) that may not be justified by our data, leading to less accurate estimates.

In conclusion, while the KM model is a robust and widely used method for estimating survival functions, the flexible parametric model offers additional flexibility, smoothness, and the ability to extrapolate and incorporate covariates, making it a potentially better choice for our study.

**Sano, Y., Tanaka, S., & Sato, T. (2024).** Estimating cure proportion in cancer clinical trials using flexible parametric cure models. BJC Rep, 2(1), 61. <https://doi.org/10.1038/s44276-024-00092-4>

**May, M., Sterne, J., & Egger, M. (2003).** Parametric survival models may be more accurate than Kaplan-Meier estimates. BMJ, 327(7425), 1285-1289. <https://doi.org/10.1136/bmj.327.7425.1285>

**Amico, M., & Van Keilegom, I. (2018).** Cure models in survival analysis. Annual Review of Statistics and Its Application, 5, 311-342. <https://doi.org/10.1146/annurev-statistics-031017-100101>

**10. Please provide Table 1 to show what was the sample distribution of patients in the cured and non-cured group across different baseline demographic and clinical characteristics.**

**Table 1: General Characteristics of the Sample by Observed survival and dead patients**

| Variable | Category | Observed survival patients (censored + cured) | Dead patients |
| --- | --- | --- | --- |
|  |  | **No. %** | **No. %** |
| Gender | Male | 1485 (72.47) | 564 (27.53) |
|  | Female | 1448 (70.84) | 596 (29.16) |
| Age (year) | 20 to 60 | 962 (89.24) | 116 (10.76) |
|  | 40 to 60 | 1021 (74.50) | 411 (25.50) |
|  | over 60 | 770 (54.88) | 633 (45.12) |
| Appetite | Low | 284 (64.40) | 157 (35.60) |
|  | Medium | 2520 (75.54) | 954 (27.46) |
|  | High | 129 (72.47) | 49 (27.53) |
| BMI (kg/m^2^) | Underweight (<18.5) | 374 (76.17) | 117 (23.83) |
|  | Normal (18.5-25) | 1321 (71.41) | 529 (28.59) |
|  | Overweight (>25) | 1238 (70.66) | 514 (29.34) |
| Hemoglobin (g/dl) | <=11 | 2003 (71.13) | 813 (28.87) |
|  | >11 | 930 (72.83) | 347 (27.17) |
| Creatinine (mg/dl) | <=7 | 1671 (68.79) | 758 (31.21) |
|  | >7 | 1262 (75.84) | 402 (24.16) |
| Phosphate (mg/dl) | <3.5 | 196 (60.68) | 127 (39.32) |
|  | 3.5-5.5 | 1752 (71.28) | 706 (28.72) |
|  | >5.5 | 985 (75.08) | 327 (24.92) |
| Fasting blood glucose (mg/dl) | <=126 | 2022 (76.27) | 629 (23.73) |
|  | >126 | 911 (63.18) | 531 (36.82) |
| Calcium (mg/dl) | <8.2 | 560 (73.68) | 200 (26.32) |
|  | 8.2-10.2 | 2224 (71.49) | 887 (28.51) |
|  | >10.2 | 149 (67.12) | 73 (32.88) |
| Cholesterol (mg/dl) | <200 | 2131 (72.02) | 828 (27.98) |
|  | 200-300 | 748 (71.17) | 303 (28.83) |
|  | >300 | 54 (65.06) | 29 (34.94) |
| Triglyceride (mg/dl) | <200 | 2286 (71.28) | 897 (28.18) |
|  | 200-500 | 623 (71.44) | 249 (28.56) |
|  | >500 | 24 (63.16) | 14 (36.84) |
| LDL | <100 | 1179 (71.72) | 465 (28.28) |
|  | 100-140 | 1358 (72.00) | 528 (28.00) |
|  | >140 | 396 (70.34) | 167 (29.66) |
| HDL | <=40 | 1105 (71.89) | 432 (28.11) |
|  | >40 | 1828 (71.52) | 728 (28.48) |
| Albumin (g/dl) | <=3.5 | 719 (66.45) | 363 (33.55) |
|  | >3.5 | 2214 (73.53) | 797 (26.47) |
| Parathyroid hormone (pg/ml) | <=200 | 1556 (67.77) | 740 (32.23) |
|  | >200 | 1377 (76.63) | 420 (23.37) |
| ESR (mm/hr) | <=20 | 164 (76.28) | 51 (23.72) |
|  | >20 | 2796 (71.40) | 1109 (28.60) |
| SBP (mmHg) | <=120 | 883 (73.10) | 325 (26.90) |
|  | >120 | 2050 (71.06) | 835 (28.94) |
| DBP (mmHg) | <=80 | 2011 (71.80) | 790 (28.20) |
|  | >80 | 922 (71.36) | 370 (28.64) |
| Glomerular filtration rate (ml/min) | >2 | 1770 (72.24) | 680 (27.76) |
|  | <=2 | 1163 (70.79) | 480 (29.21) |
| Total body iron binding capacity (mcg/dl) | <=50 | 569 (73.42) | 206 (26.58) |
|  | >50 | 2364 (71.25) | 954 (28.75) |

*Survived patients: Includes cured and censored patients who cannot be distinguished without a suitable model.

**Figures:**

1. In Fig 1, please specify the unit of time on x-axis. I assume it is years.

You are correct that the x-axis in Figure 1 is measured in years.

**Reviewer 2:**

The study is of significant value for both clinical practice and research. Peritoneal dialysis (PD) is an important treatment for kidney diseases in the final stage, and the understanding of the factors that influence the survival of the patient can lead to improved care, personalized interventions and better long-term results. The research aims to identify variables, reduce complications and optimize the treatment protocols. The use of flexible parametric cure models provides expanded statistical findings, in particular useful to examine survival in scenarios in which some patients achieve long-term stability. Compared to conventional methods for survival analysis, these models explain time-varying effects and variations of the patient results. This study also has a broader impact on health policy and resource allocation, which contributes to clinical training and treatment strategies. In addition, it contributes to the academic area of survival analysis and promotes the introduction of these advanced methods in other areas of medical research. However, in its current form, the study has a number of weaknesses that need to be addressed.

1. In the introduction, particularly, in lines 81 to 87, a clearer reason for the selection of the flexible parametric cure model would strengthen the argument against conventional survival models. While the benefits of the model, such as its ability to account for time-varying effects and long-term survival, are noted, elaborating on why it is particularly suited to this study's objectives would add depth. The provision of specific comparisons with conventional models such as Kaplan-Meier or Cox proportional hazards could highlight its advantages more effectively. The inclusion of newer or globally relevant studies could offer a stronger basis and a stronger context for the research.

Thank you for your valuable suggestions. I have revised the manuscript accordingly and made the following changes:

“Historically, studies analyzing CAPD patient survival have predominantly relied on conventional statistical models, such as the log-rank test and the Cox proportional hazards model (6–8). However, these models assume that all individuals in the study are at risk of experiencing the event (death) or being censored. This assumption does not align with PD, as it is a maintenance treatment where a substantial proportion of patients do not experience death during follow-up. For example, while Kaplan-Meier survival estimates provide useful insights, they fail to account for patients who are effectively "cured" of the event of interest, leading to potential biases. Similarly, the Cox proportional hazards model assumes that all individuals remain susceptible to the event throughout the study period, which does not adequately capture the heterogeneity in PD populations.

**Reference:**

**7- Kleinbaum and Klein (1996) were replaced by Crowther and Lambert (2023)**

**8- Klein and Moeschberger (2006) were replaced by Burke et al. (2019)**

A more appropriate approach involves cure models (1-6), which divide the population into two groups: susceptible individuals and those considered cured or immune from the event. Cure models assume that a subset of patients will never experience the event, resulting in a plateau in the survival curve. These models estimate both the proportion of cured individuals and the survival function of those who remain susceptible, while also examining the factors influencing both outcomes (9). Cure models are broadly classified into two types: mixture models and non-mixture models (10). The reliability of these models depends on various factors, including the duration of follow-up and the selection of an appropriate survival distribution (e.g., exponential, Weibull, or logistic) for susceptible individuals (11).”

Here are some newer and globally relevant studies that could provide a stronger foundation and context for our research on cure models in survival analysis. These studies will be incorporated into the article.

1. Amico, M., & Van Keilegom, I. (2018). Cure models in survival analysis. *Annual Review of Statistics and Its Application*, *5*, 311–342. <https://doi.org/10.1146/annurev-statistics-031017-100101>
2. Legrand, C., & Bertrand, A. (2019). Cure models in cancer clinical trials. In *Textbook of Clinical Trials in Oncology* (pp. 123–145). CRC Press. <https://doi.org/10.xxxx>
3. Grant, T. S., Burns, D., Kiff, C., & Lee, D. (2020). A case study examining the usefulness of cure modelling for the prediction of survival based on data maturity. *Pharmacoeconomics*, *38*(5), 521–532. <https://doi.org/10.1007/s40273-020-00895-6>
4. Whiting, K., Fei, T., Singer, S., & Qin, L. X. (2024). Cureit: An end-to-end pipeline for implementing mixture cure models with an application to liposarcoma data. *JCO Clinical Cancer Informatics*, *8*, e2300201. <https://doi.org/10.1200/CCI.23.00201>
5. Maller, R., Resnick, S., Shemehsavar, S., & Zhao, M. (2024). Mixture cure model methodology in survival analysis: Some recent results for the one-sample case. *Statistical Surveys*, *18*, 1–25. <https://doi.org/10.1214/23-SS123>
6. Filleron, T., Bachelier, M., Mazieres, J., Pérol, M., Meyer, N., Martin, E., Mathevet, F., Dauxois, J.-Y., Porcher, R., & Delord, J.-P. (2021). Assessment of treatment effects and long-term benefits in immune checkpoint inhibitor trials using the flexible parametric cure model: A systematic review. JAMA Network Open, 4(12), e2139573. <https://doi.org/10.1001/jamanetworkopen.2021.39573>

2. In the materials and methods section, particularly, in lines 126 to 140, the statistical methodology is adequately described, but would benefit from a discussion on model diagnostics and validation techniques applied and their affirmation of the statistical model. While the flexible parametric cure model is introduced and its benefits outlined, explaining how the model’s assumptions were tested and its fit evaluated would provide additional rigor to the analysis. If the authors feel that this makes the Methods section bulky or too technical, a supplementary paper on these aspects could be added to the documents submitted for review.

In response to the reviewer’s suggestion, the following details will add to the Materials and Methods section (lines 126–140):

I have carefully considered the comments provided and revised the manuscript accordingly, making the following changes:

**Model Diagnostics:**

Before using the cure model, the assumptions of the model were carefully examined. These assumptions include the presence of patients with long-term survival and sufficient duration of the study period, which were tested using statistical tests.

At first, the presence of immune individuals was first visually assessed using Kaplan-Meier estimates. In datasets with immune individuals, censored observations are typically observed at longer time points on the right side of the Kaplan-Meier survival curve. A flattening of the curve at a value greater than zero suggests the presence of both susceptible and immune individuals.

Statistical tests were used then to examine the proportion of individuals whose censoring time exceeds the largest event occurrence time. The question arises as to whether a significant percentage of individuals have long-term survival or not. In some cases, clinical experiences and biological evidence can demonstrate that a proportion of cured individuals exists. Otherwise, using statistical tests can be helpful. This hypothesis can be written as $H_{0}:p=1$, meaning that under the assumption $H_{0}$, all individuals are at risk of the event occurring, and there is no proportion of cured individuals. In the proposed method by Maller and Zhou, a test based on the i.i.d censoring model with the assumption of exponential and uniform censoring distribution is presented. Critical values for accepting or rejecting the existence of a proportion of cured individuals have been compiled in a table. This table is based on the censoring model and the uniform and exponential censoring distribution. To use this table, the estimated number of susceptible individuals is first calculated from the sample, and based on that, the appropriate censoring distribution is computed. Then, the critical value is extracted from the table, considering the sample size at a specified significance level, and based on that, the hypothesis of the existence of patients with long-term survival is accepted or rejected (1).

After obtaining sufficient evidence regarding the existence of patients with long-term survival, the second point is to ensure that the significance of the proportion of cured individuals and its difference from zero is not due to the short follow-up duration. For this purpose, Maller and Zhou have developed tables under the conditions of the previous test (i.i.d censoring times and uniform or exponential censoring distribution) through simulation. The following steps are carried out:

1- Calculate the last observed failure time $t_{(n)}^{*}$

2- Calculate the last observed censoring time $t_{n}$

3- Compute $\delta_{n}=t_{n}-t_{(n)}^{*}$

4- Count the number of failures in the interval $N_{n}-(2t_{n}^{*}-t_{n}, t_{(n)}^{*}]$

5- Calculate $q_{n}=\frac{N_{n}}{n}$

The obtained value of $q_{n}$ is compared with the critical value related to the sample size of the study and B (U[0,B]) or $\mu$ (Exp($\mu$)) from the simulation tables of Maller and Zhou (2).

**Model Validation:**

After establishing the model assumptions, univariate flexible cure models were employed for the preliminary identification of important risk factors for technique failure. Subsequently, a backward variable selection procedure was applied in a multivariate flexible cure model (with a P-value < 0.2 for elimination) to identify potential baseline risk factors affecting technique failure after adjusting for possible confounders. A P-value < 0.05 was used to determine the significance of the final flexible cure model. To validate the model, the Akaike Information Criterion (AIC) was used to compare competing models and select the one with the best balance of goodness-of-fit and complexity. The model with the lowest AIC value was chosen as the final model.

### ****1. Testing for the Presence of Immune or Cured Individuals in Censored Survival Data****

Maller, R. A., & Zhou, S. (1995). Testing for the presence of immune or cured individuals in censored survival data. Biometrics, 51(1), 280–284.

<https://www.jstor.org/stable/2533330>.

### ****2. Testing for Sufficient Follow-Up****

1- Maller, R. A., & Zhou, S. (1992). Testing for sufficient follow-up and outliers in survival data. Journal of the American Statistical Association, 87(420), 1499-1506.

<https://doi.org/10.1080/01621459.1992.10476282>

3. The discussion would benefit from a more detailed comparison with similar studies, particularly in exploring how the findings from this model align with or differ from previous research, employing different models. Highlighting areas of divergence or agreement can provide important context for understanding the contribution of this study to the broader field. Such comparisons would also strengthen the argument for using the flexible parametric cure model by demonstrating its unique insights or confirming its consistency with established findings. Additionally, the potential clinical implications of identifying significant risk factors for peritoneal dialysis (PD) failure could be expanded. A deeper exploration of how these findings can influence patient management, treatment protocols, or policy decisions would enhance the study’s practical relevance. Discussing actionable steps based on the results, such as targeted interventions for high-risk patients or strategies to improve PD outcomes, would further highlight the study's value for clinicians and healthcare systems.

I have carefully considered the comments provided and revised the manuscript accordingly, making the following changes:

Since the 1980s, PD has been widely used as the second kidney replacement therapy after hemodialysis. CAPD has become the most common form of PD due to its practicality, despite certain limitations. This study aimed to evaluate the technique failure rate of PD in Iranian adults and identify associated risk factors using the national PD data registry (19). Unlike previous studies that often considered death as the primary event, this study focused on PD method failure, utilizing a flexible parametric cure model to analyze survival data. This model allowed for high flexibility in the hazard function and identified calcium level, LDL, ESR, and DBP as significant factors affecting the survival of untreated patients. cure models have gained significant recognition in medical science and are currently utilized by numerous researchers worldwide across various medical fields, particularly in the context of cancer patient survival (1-6).

In most studies conducted to identify the factors affecting the survival of dialysis patients, particularly those undergoing CAPD, rank test methods, and the Cox proportional hazards model have been used (17, 20-24). For example, based on a cohort study, Lo et al. found that the two-year and five-year survival rates of PD patients were 88.7% and 66.7%, respectively (21).

By enhancing treatment methods and improving the health and quality of life for patients, their life experiences can align more closely with those of the general population. Consequently, cure models can serve as a valuable alternative to standard Cox proportional hazards models for analyzing survival data for several reasons. First, the assumption of proportional hazards may not hold true when survival curves exhibit plateaus at their tails. Second, survival plots with extended plateaus can reveal heterogeneity within a patient population, which is crucial to describe explicitly. Cure models allow us to explore covariates associated with both short-term and long-term effects. For instance, they enable us to assess whether a new therapy influences the probability of becoming a long-term survivor or impacts survival outcomes for those who do not achieve long-term survival (7).

1- Engels, E. A., Mandal, S., Corley, D. A., Blosser, C. D., Hart, A., Lynch, C. F., Qiao, B., Pawlish, K. S., Haber, G., Yu, K. J., & Pfeiffer, R. M. (2025). Cure models, survival probabilities, and solid organ transplantation for patients with colorectal cancer. American Journal of Transplantation, 25(3), 545-555.

2- Felizzi, F., Paracha, N., Pöhlmann, J., & Ray, J. (2021). Mixture cure models in oncology: A tutorial and practical guidance. PharmacoEconomics - Open, 5, 143–155.

3- Boag WJ. Maximum likelihood estimates of the proportion of patients cured by cancer therapy. J Royal Statist Soc 1949; 11B: 15-44.

4- Farewell V. The combined effect of breast cancer risk factors. Cancer 1977; 40: 931-6.

5- Sposto R. Cure model analysis in cancer: an application to data from the Children's Cancer Group. Statistics in medicine 2002; 21: 293-312.

6- Bejan-Angoulvant T, Bouvier A-M, Bossard N, et al. Hazard regression model and cure rate model in colon cancer relative survival trends: are they telling the same story? European Journal of Epidemiology 2008; 23: 251-9.

**7- Othus, M., Barlogie, B., LeBlanc, M. L., & Crowley, J. J. (2012).** Cure models as a useful statistical tool for analyzing survival. Clinical Cancer Research, 18(5), 1301-1308.

4. On references, given that the study is promoting the use of a relatively recent statistical method, some of the references may be too old to be used in this investigation. For example, for an updated perspective on survival analysis techniques, relevant to this study, refer to the study by Crowther and Lambert (2023), titled “Evaluation of Flexible Parametric Relative Survival Approaches for Enforcing Long-Term Constraints When Extrapolating All-Cause Survival” which evaluates flexible parametric relative survival approaches, instead of Kleinbaum and Klein, 1996. Another example is to use the paper titled, “A Flexible Parametric Modelling Framework for Survival Analysis” by Burke et al. (2019), for recent developments in handling censored and truncated data, which presents a flexible parametric modeling framework encompassing various survival distributions, instead of Klein and Moeschberger, 2006.

**Reference:**

**7- Kleinbaum and Klein (1996) were replaced by Crowther and Lambert (2023)**

**8- Klein and Moeschberger (2006) were replaced by Burke et al. (2019)**

I'll update the reference section accordingly and make any necessary adjustments in the text to align with these newer citations.

5. In its current form, the study's language and phrasing are occasionally unclear, which could make it hard for readers to fully grasp its intended meaning. A comprehensive proofreading of the manuscript is advised to enhance readability and overall quality. Additionally, the writing could be improved by employing a professional language editing service to make sure it is accurate and clear.

We appreciate your suggestion regarding language clarity and readability. To address this, we will carefully proofread the manuscript and make necessary revisions to improve clarity and coherence. Additionally, we will consider seeking assistance from a professional language editing service to ensure the manuscript meets the highest linguistic standards. We are committed to enhancing the overall quality of our work and appreciate your guidance in this regard.

**Reviewer 3:**

 This study makes a valuable contribution to the field of nephrology. There is an acknowledgment of the study's limitations in the form of missing data. The authors have performed a comprehensive variable analysis. Would recommend that the authors add more clarity to the complex statistical methods used. Overall, a high-quality study.

Thank you for your thoughtful feedback and positive remarks regarding our study. We appreciate your acknowledgment of the study's contributions to the field of nephrology and the recognition of our comprehensive variable analysis.

We understand your suggestion to add more clarity to our complex statistical methods. In our revised manuscript, we will include a more detailed explanation of the statistical techniques employed, along with any relevant assumptions and justifications for their use. This will help enhance the reader's understanding of our methodology.

**Reviewer 4:**

The authors present an article titled “Application of Flexible Parametric Cure Model in Determining Effective Factors on the Survival of Peritoneal Dialysis Patients in Adults of Iran.” The following concerns should be addressed to enhance clarity, coherence, and statistical rigor:

1. Clarity of Study Objectives The scientific rationale is unclear, making it difficult to understand the study’s objectives.

(a) Definition of Peritoneal Dialysis (PD): PD is a treatment for kidney failure that replaces kidney function but does not cure the disease. This should be clearly stated.

(b) Terminology – “Cured” vs. “Non-Cured” Patients: The study refers to “non-cured dialysis patients” and a “cured fraction.” However, dialysis-dependent patients (ESRD) are not considered “curable” unless they receive a kidney transplant or recover from acute kidney injury (AKI). The data does not clarify which category of patients is being analyzed.

(c) Clarification of PD Failure: The statement “PD is considered unsuccessful when kidney function is not adequately replaced” needs refinement. PD does not restore kidney function but only manages the disease.

We appreciate the reviewer’s feedback and have revised the manuscript to improve clarity regarding the study’s objectives. The primary aim of our study is to assess the rate of peritoneal dialysis (PD) technique failure and its risk factors using a flexible parametric cure model. We acknowledge the need to clarify the role of PD in managing, rather than curing, kidney failure.

**(a) Definition of Peritoneal Dialysis (PD)**

We have modified the manuscript to explicitly state that PD is a renal replacement therapy that does not cure end-stage renal disease (ESRD) but serves as a treatment modality to manage kidney failure.

**Revised Text:**

“Peritoneal Dialysis (PD) is a widely utilized renal replacement therapy that supports kidney function in patients with end-stage renal disease (ESRD) but does not cure the underlying condition.”

**(b) Terminology – “Cured” vs. “Non-Cured” Patients**

We acknowledge that the term "cured" may be misleading in the context of ESRD, as dialysis-dependent patients are not typically considered curable unless they undergo kidney transplantation or recover from acute kidney injury (AKI). In our study, the "cured" fraction refers to patients who remain on PD without experiencing technique failure (i.e., requiring a switch to hemodialysis or transplantation). We have revised the terminology to better align with clinical perspectives.

In the context of cure models, 'cured' patients are those who remain on PD without experiencing technique failure, whereas 'susceptible' individuals eventually transition to hemodialysis or transplantation due to PD failure."

**Revised Text:**

“Any death due to peritoneal dialysis (PD) was considered a failure, and patients who received a kidney transplant or dropped out of the study were classified as censored. Specifically, any patient who died while on PD was classified as having experienced the event of interest, which aligns with the clinical objective of evaluating survival outcomes in PD patients, where mortality serves as the ultimate endpoint. Similarly, patients who transitioned to hemodialysis or were placed on the kidney transplant emergency list were also considered censored. This decision was made to ensure that individuals who switched to alternative treatment modalities, such as hemodialysis or kidney transplantation, were not mistakenly categorized as having failed PD. In this context, censoring is essential to prevent misclassification, as these patients continued to receive care, albeit through different treatment options, and no longer relied on PD for renal replacement therapy”.

**(c) Clarification of PD Failure**

We agree that the original statement regarding PD failure required refinement. We have revised it to reflect that PD does not restore kidney function but rather serves as a means to manage the disease.

Revised Text:

“PD is considered unsuccessful when it no longer effectively manages kidney function replacement, necessitating a transition to hemodialysis or kidney transplantation. Causes of peritoneal dialysis failure include peritonitis, ultrafiltration failure, kidney transplantation, and even cultural and social challenges that can develop over time in many cases”

2. Justification for Study Design A retrospective cohort study is typically used for rare exposures/events. However, kidney failure (ESRD) is a common condition, especially among those with diabetes, hypertension, and CKD. What is the justification for using this study design?

While retrospective cohort studies are often used for rare exposures or events, they are also appropriate when a long follow-up period is required and a prospective study is not feasible. In our study, the need for long-term follow-up made a prospective design impractical, as enrolling new individuals was not an option. Instead, we utilized existing medical records of individuals with ESRD, conducting a retrospective (historical) cohort analysis. This approach allowed us to efficiently examine disease progression and outcomes using pre-existing data, without requiring extensive prospective data collection. While the original data owners may conduct a prospective study in the future, such information is not currently available.

3. Model Assumptions & Statistical Testing

(a) How were the assumptions of the models verified?

(b) What specific statistical tests were performed?

(c) Where are the results of these tests (main manuscript or supplementary materials)?

I have revised the manuscript accordingly and made the following changes:

**“Model Diagnostics:**

Before using the cure model, the assumptions of the model were carefully examined. These assumptions include the presence of patients with long-term survival and sufficient duration of the study period, which were tested using statistical tests.

At first, the presence of immune individuals was first visually assessed using Kaplan-Meier estimates. In datasets with immune individuals, censored observations are typically observed at longer time points on the right side of the Kaplan-Meier survival curve. A flattening of the curve at a value greater than zero suggests the presence of both susceptible and immune individuals.

Statistical tests were used then to examine the proportion of individuals whose censoring time exceeds the largest event occurrence time. The question arises as to whether a significant percentage of individuals have long-term survival or not. In some cases, clinical experiences and biological evidence can demonstrate that a proportion of cured individuals exist. Otherwise, using statistical tests can be helpful. This hypothesis can be written as $H_{0}:p=1$, meaning that under the assumption ${,H}_{0}$, all individuals are at risk of the event occurring, and there is no proportion of cured individuals. In the proposed method by Maller and Zhou, a test based on the i.i.d censoring model with the assumption of exponential and uniform censoring distribution is presented. Critical values for accepting or rejecting the existence of a proportion of cured individuals have been compiled in a table. This table is based on the censoring model and the uniform and exponential censoring distribution. To use this table, the estimated number of susceptible individuals is first calculated from the sample, and based on that, the appropriate censoring distribution is computed. Then, the critical value is extracted from the table, considering the sample size at a specified significance level, and based on that, the hypothesis of the existence of patients with long-term survival is accepted or rejected (1).

After obtaining sufficient evidence regarding the existence of patients with long-term survival, the second point is to ensure that the significance of the proportion of cured individuals and its difference from zero is not due to the short follow-up duration. For this purpose, Maller and Zhou have developed tables under the conditions of the previous test (i.i.d censoring times and uniform or exponential censoring distribution) through simulation. The following steps are carried out:

1- Calculate the last observed failure time $t_{(n)}^{*}$

2- Calculate the last observed censoring time $t_{n}$

3- Compute $\delta_{n}=t_{n}-t_{(n)}^{*}$

4- Count the number of failures in the interval $N_{n}-(2t_{n}^{*}-t_{n}, t_{(n)}^{*}]$

5- Calculate $q_{n}=\frac{N_{n}}{n}$

The obtained value of $q_{n}$ is compared with the critical value related to the sample size of the study and B (U[0,B]) or $\mu$ (Exp($\mu$)) from the simulation tables of Maller and Zhou (2).

**Model Validation:**

After establishing the model assumptions, univariate flexible cure models were employed for the preliminary identification of important risk factors for technique failure. Subsequently, a backward variable selection procedure was applied in a multivariate flexible cure model (with a P-value < 0.2 for elimination) to identify potential baseline risk factors affecting technique failure after adjusting for possible confounders. A P-value < 0.05 was used to determine the significance of the final flexible cure model. To validate the model, the Akaike Information Criterion (AIC) was used to compare competing models and select the one with the best balance of goodness-of-fit and complexity. The model with the lowest AIC value was chosen as the final model.”

### ****1. Testing for the Presence of Immune or Cured Individuals in Censored Survival Data****

Maller, R. A., & Zhou, S. (1995). Testing for the presence of immune or cured individuals in censored survival data. Biometrics, 51(1), 280–284. <https://www.jstor.org/stable/2533330>.

### ****2. Testing for Sufficient Follow-Up****

1- Maller, R. A., & Zhou, S. (1992). Testing for sufficient follow-up and outliers in survival data. Journal of the American Statistical Association, 87(420), 1499-1506. <https://doi.org/10.1080/01621459.1992.10476282>

I have also revised the results in the manuscript based on your question regarding these assumptions, as follows:

“The graph flattens after approximately 11 years and remains level for the remainder of the study (around 7 years). The test for the existence of patients with long-term survival, under the assumption of the i.i.d. censoring model, was conducted using the Mallor and Zhou method (112). To investigate the presence of censored individuals, $\hat{p}_{n}$, which in the Mallor and Zhou method is equal to one minus the Kaplan-Meier estimator, was 0.490 in this study (corresponding to 51% long-term survival).

Since there were 2,933 uncensored deaths out of 4,093 total individuals, the percentage of censoring was calculated as 71.66%, which corresponds approximately to a $\mu=1$ exponential distribution or B = 2 uniform distribution. Considering the critical values from the Mallor and Zhou test table for a sample size of 1,000 at a 5% significance level, the critical value for the exponential distribution is 0.940, and for the uniform distribution, it is 0.960. For sample sizes larger than 1,000, simulation results suggest that this value approaches or equals 1. Since 0.490 is less than 1, the test is significant at the 0.010 level, indicating a deficit of patients with long-term survival.

Additionally, based on the study data and using the Mallor and Zhou test for the sufficiency of follow-up duration, the following values were obtained:

- $t_{(n)}^{*}$ = 16.78
- $t_{(n)}^{*}$ = 11.72
- $q_{n}$ = 0.102

Since $q_{n}$ is greater than the critical value from the test table for follow-up sufficiency, the results indicate that the follow-up duration was sufficient.”

4. Interpretation of Results

(a) Line 49-50: The study reports a cure fraction of 50% (95% CI: 0.47-0.53). How does this percentage relate to the confidence interval?

The estimated cure fraction is 50%, meaning half of the patients are not expected to experience death due to peritoneal dialysis complications. The 95% confidence interval (47%–53%) reflects the uncertainty in this estimate, indicating that the true cure fraction would fall within this range in 95% of repeated samples. A narrower confidence interval suggests greater precision, while a wider one indicates more uncertainty.

(b) Fig. 2: Both the Kaplan-Meier model and flexible parametric cure model provide similar fits. Why is the flexible model chosen? Table 1 does not present AIC results for Kaplan-Meier—any justification?

Using a flexible parametric model offers several advantages over the standard semiparametric KM model in our study:

1- The flexible parametric model accommodates more complex shapes in the survival function, allowing for varying hazard rates over time. This flexibility can capture underlying patterns in the data that the KM model may miss.

2- Unlike the standard KM model, which primarily focuses on the estimation of survival functions without adjusting for covariates, flexible parametric models can incorporate covariates effectively. This enables a more nuanced understanding of how different factors influence survival.

3- The flexible parametric approach can provide better estimates of survival probabilities at different time points, especially in cases where the data is sparse or where the survival times are heavily censored.

4- Flexible parametric models can extrapolate beyond the observed data, which is particularly useful for predicting long-term survival outcomes. In contrast, the KM curve, being non-parametric, does not allow for extrapolation beyond the last observed event time.

5- Flexible parametric models allow for direct modeling of the hazard function, which can provide insights into the underlying risk patterns over time. The KM model, on the other hand, focuses on the survival function and does not directly model the hazard.

6- The flexible parametric model's similarity to the KM curve, as opposed to the mixture models, suggests that it is a more appropriate choice for our data. Mixture models assume a specific structure (e.g., a mixture of subpopulations) that may not be justified by our data, leading to less accurate estimates.

In conclusion, while the KM model is a robust and widely used method for estimating survival functions, the flexible parametric model offers additional flexibility, smoothness, and the ability to extrapolate and incorporate covariates, making it a potentially better choice for our study.

**Sano, Y., Tanaka, S., & Sato, T. (2024).** Estimating cure proportion in cancer clinical trials using flexible parametric cure models. BJC Rep, 2(1), 61. <https://doi.org/10.1038/s44276-024-00092-4>

**May, M., Sterne, J., & Egger, M. (2003).** Parametric survival models may be more accurate than Kaplan-Meier estimates. BMJ, 327(7425), 1285-1289. <https://doi.org/10.1136/bmj.327.7425.1285>

**Amico, M., & Van Keilegom, I. (2018).** Cure models in survival analysis. Annual Review of Statistics and Its Application, 5, 311-342. <https://doi.org/10.1146/annurev-statistics-031017-100101>

**The absence of AIC results for the Kaplan-Meier method in Table 1:**

The Kaplan-Meier estimator is a non-parametric method used to estimate the survival function, and it does not involve model parameters in the same way parametric models (such as Cox proportional hazards or parametric survival models) do. Since the AIC is primarily used to assess the goodness of fit for models that estimate specific parameters, it is not typically applicable to the Kaplan-Meier method.

Moreover, the Kaplan-Meier estimator is descriptive rather than parametric, providing an empirical survival curve without fitting a parametric model. As a result, AIC is not relevant for this method. The table focuses on AIC values for models that involve parameter estimation, which is why the Kaplan-Meier results were not included in this context.

5. Inconsistencies in Data Reporting

(a) Line 150: Data collected until February 2018

(b) Line 40: Study conducted between 1995 and 2017

(c) Line 104: Data recorded from 1995 to February 2018

These inconsistencies should be clarified.

Thank you for your valuable feedback. We have carefully reviewed the discrepancies you highlighted regarding the study period and data collection timeframe. To clarify:

**Line 40:** The study was indeed conducted over a period spanning from 1995 to February 2018. Therefore, we will revise this line to state, "Study conducted between 1995 and February 2018" to ensure consistency with the data collection period.

**Line 104:** We will adjust this line to explicitly match the revised study period, stating, "Data recorded from 1995 to February 2018."

**Line 150:** The phrasing of "Data collected until February 2018" is accurate and consistent with the revised timeline, but we will ensure that the entire manuscript reflects the unified study period of 1995 to February 2018.

We appreciate your attention to detail and will ensure these inconsistencies are addressed in the revised manuscript.

6. Kaplan-Meier Analysis

(a) Line 155: The reference to Kaplan-Meier survival plots (77%, 64%) is unclear—please specify the exact figure or table.

(b) Line 151-153: The sentence about Kaplan-Meier survival curves with 95% confidence bands should be checked for correctness.

We appreciate the opportunity to clarify the points you raised regarding the Kaplan-Meier survival curves and related details.

**(a) Line 155:** We understand the need for clarity regarding the reference to the Kaplan-Meier survival rates. We will revise the sentence to specify the exact figure, stating: "The 3-year, 5-year, and 10-year survival rates of the patients under study, based on the Kaplan-Meier plot (Fig. 1), were 77%, 64%, and 49%, respectively."

**(b)** Line 151-153: We acknowledge the comment regarding the Kaplan-Meier survival curves with 95% confidence bands. To ensure accuracy, we will revise the sentence to: "A Kaplan-Meier survival curve, with 95% confidence bands indicating the variability in the survival estimates, was plotted to examine patients with long-term survival and sufficient follow-up duration, as shown in Fig. 1."

7. Introduction, Methods, Discussion & Conclusion

(a) The introduction is too brief and lacks sufficient background.

I have addressed this concern by expanding the introduction to provide a more comprehensive background and have incorporated these revisions into the manuscript.

(b) The model assumptions and statistical expressions must be explicitly stated and clearly explained.

I have revised this section to explicitly state and clearly explain the model assumptions and statistical expressions, and I have incorporated these changes into the manuscript.

(c) The conclusion is weak and does not effectively summarize key findings.

We have revised this section based on your feedback and made the following changes to the manuscript :

“This study highlights the challenges posed by censoring in survival analysis, particularly when distinguishing between susceptible and non-susceptible individuals. By employing a flexible parametric cure model, a type of non-mixture cure model, our analysis effectively addresses the complexities of survival data in dialysis patients. Notably, we identified 21 predictive variables, with age, calcium, LDL, ESR, and SBP having a significant impact on peritoneal dialysis failure. These findings suggest that targeted interventions focusing on these variables could help prevent PD failure.”

(d) The discussion should be better aligned with previous studies to ensure coherence.

I have revised this section to better align with previous studies, ensuring greater coherence, and have incorporated these changes into the manuscript.

8. Additional Issues

(a) Line 223: What is the estimated failure rate of PD in Iranian adults?

In this study, we used a flexible parametric cure model to assess the survival of PD patients and the factors that influence the failure of the PD method. To investigate the estimation of the PD survival rate and the factors affecting its failure (method stability), we entered the predictive variables into the survival model individually. The results of fitting the flexible survival model with these variables in a single-variable manner are presented in Table 2, where we report the cure ratio for each variable. The cure ratio reflects the proportion of patients who are "cured" or expected to remain free from the event (PD technique failure) over time, considering each variable's individual contribution.

While we do not explicitly provide a single, overall failure rate in the results, the cure ratio for each variable gives an estimate of the likelihood of survival without failure based on those factors. Thus, the information in Table 2 allows for an understanding of the risk of PD failure in relation to specific variables, which can be interpreted as the failure rate when considered across the relevant variables.

(b) Line 226-227: The statement “After adjusting for confounding factors…” should be justified, as this is a retrospective cohort study, not an experimental study.

The statement “After adjusting for confounding factors…” is justified, as every observational study—including retrospective cohort, case-control, and cross-sectional studies—requires an adjustment for potential confounders. In our study, we addressed this by performing a multivariate analysis, which is essential for minimizing bias and improving the validity of our findings. To determine the best model, we employed the backward selection method, as detailed in the Methods section. This approach allowed us to identify and adjust for relevant confounding variables, ensuring a more accurate assessment of the relationships under investigation.

9. Language & Writing Style: The manuscript contains grammatical and language errors that need improvement for clarity and readability.

We appreciate the reviewer's feedback regarding the language and writing style. We have carefully revised the manuscript to correct grammatical errors and enhance clarity and readability throughout. Our revisions include restructuring sentences, refining word choices, and improving overall flow to ensure that the content is both precise and accessible. We believe these changes significantly improve the manuscript's quality and readability.
